# Unleashing the power of meta-threading for evolution/structure-based function inference of proteins

**DOI:** 10.3389/fgene.2013.00118

**Published:** 2013-06-19

**Authors:** Michal Brylinski

**Affiliations:** ^1^Department of Biological Sciences, Louisiana State UniversityBaton Rouge, LA, USA; ^2^Center for Computation and Technology, Louisiana State UniversityBaton Rouge, LA, USA

**Keywords:** protein function inference, template-based modeling, protein meta-threading, ligand-binding, metal-binding, iron/sulfur-binding, protein-protein interactions, protein-DNA interactions

## Abstract

Protein threading is widely used in the prediction of protein structure and the subsequent functional annotation. Most threading approaches employ similar criteria for the template identification for use in both protein structure and function modeling. Using structure similarity alone might result in a high false positive rate in protein function inference, which suggests that selecting functional templates should be subject to a different set of constraints. In this study, we extend the functionality of *e*Thread, a recently developed approach to meta-threading, focusing on the optimal selection of functional templates. We optimized the selection of template proteins to cover a broad spectrum of protein molecular function: ligand, metal, inorganic cluster, protein, and nucleic acid binding. In large-scale benchmarks, we demonstrate that the recognition rates in identifying templates that bind molecular partners in similar locations are very high, typically 70–80%, at the expense of a relatively low false positive rate. *e*Thread also provides useful insights into the chemical properties of binding molecules and the structural features of binding. For instance, the sensitivity in recognizing similar protein-binding interfaces is 58% at only 18% false positive rate. Furthermore, in comparative analysis, we demonstrate that meta-threading supported by machine learning outperforms single-threading approaches in functional template selection. We show that meta-threading effectively detects many facets of protein molecular function, even in a low-sequence identity regime. The enhanced version of *e*Thread is freely available as a webserver and stand-alone software at http://www.brylinski.org/ethread.

## INTRODUCTION

Currently, the most accurate and consequently the most widely used methods for protein structure and function prediction build on homology, i.e., they use information educed from related proteins. As demonstrated in the recent Critical Assessment of Protein Structure Prediction (CASP) experiment, the top performing groups in tertiary structure and function prediction categories use various template-based methods (Moult et al., 2009). Typically, the first step in comparative protein structure modeling or function inference is the identification of suitable templates in available databases, such as the Protein Data Bank (PDB; Berman et al., 2000). Here, the simplest approaches detect template proteins using sequence comparisons; however, these methods are generally limited to the high levels of sequence identity (Rost, 2002).

To address this issue, a number of methods have been developed to search for low-sequence identity templates that can be used to construct the structural model of a target protein or to infer its molecular function. Nevertheless, because of the complex and equivocal relations between protein sequence, structure, and function, template-based modeling in the “twilight zone” of sequence similarity (Rost, 1999) may result in a high false positive rate. This problem can be addressed by introducing various scoring functions and filters. For example, a sequence profile score, pairwise interaction potential, environmental fitness, secondary structure compatibility and their linear combinations are popular scoring functions widely used in threading and fold recognition. To provide templates for protein structure prediction, many methods, such as SP3 (Zhou and Zhou, 2005) and SPARKS2 (Zhou and Zhou, 2005), first apply a combined scoring function to assign the score to each structure in the template library and then use a *Z*-score filter to identify these templates that are likely structurally similar to the target. Similar criteria are commonly used for the template identification in protein function prediction. For example, to collect functional templates, FINDSITE (Brylinski and Skolnick, 2008) and FINDSITE-metal (Brylinski and Skolnick, 2011) use a threading *Z*-score of ≥4, a threshold that is also used in the detection of structural templates in TASSER (Zhang and Skolnick, 2004b). A similar method, @TOME-2, employs meta-threading to detect template proteins, which are subsequently used in both structure modeling and function inference (Pons and Labesse, 2009). Other approaches such as 3DLigandSite (Wass et al., 2010) or I-TASSER (Roy et al., 2010) use structural alignments to detect ligand-bound templates for function prediction.

It has been demonstrated that using structure similarity alone may result in a high false positive rate in protein function inference (Brylinski and Skolnick, 2010). Historically, template identification algorithms, such as threading or fold recognition, were designed to detect structurally similar templates for protein structure prediction. Therefore, template selection by structure-driven threading or structure alignment may provide false information on protein function. Recently, we developed *e*Thread, an accurate meta-threading procedure for the identification of structurally related templates for the template-based modeling of proteins (Brylinski and Feinstein, 2012; Brylinski and Lingam, 2012). Here, we extend its functionality to also include the optimal selection of functional templates, which cover a broad range of protein function: ligand, metal, inorganic cluster, protein, and nucleic acid binding. We show that state-of-the-art threading algorithms effectively detect many aspects of protein molecular function using low-homology templates, thus should provide practical assistance to evolution/structure-based function inference of proteins.

## METHODS

### DATASETS

Five datasets were compiled in this study, which comprise ligand-, metal-, and iron/sulfur-binding proteins as well as protein–protein and protein–DNA macromolecular complexes. Metal- and Fe/S-binding proteins were directly identified in the PDB (Berman et al., 2000). Following metals were included in the dataset: Ca, Co, Cu, Fe, Mg, Mn, and Zn. Iron–sulfur clusters are made up of at least two iron and two sulfur atoms. The set of protein–ligand complexes was obtained from the Protein-Small Molecule Database (Wallach and Lilien, 2009), which provides a convenient resource for studies focusing on protein–small molecule interactions. Small organic compounds non-covalently bound to proteins and composed of 7–60 heavy atoms were included in the dataset. Protein–protein and protein–DNA complexes were downloaded from the PISA database of macromolecular assemblies (Krissinel and Henrick, 2007). For protein–protein complexes, the minimum number of interfacial residues was set to 20, for protein–DNA assemblies, only DNA strands with at least 10 nucleotides are considered. In each dataset, the redundancy was removed at 40% pairwise sequence identity using PISCES (Wang and Dunbrack, 2003). Furthermore, we included only proteins 50–600 residues in length. This procedure resulted in 6,895, 6,610, 209, 8,155, and 440 ligand-, metal-, Fe/S-, protein-, and DNA-binding proteins, respectively. The lists of benchmarking proteins are provided as Supplementary Materials.

### FUNCTIONAL TEMPLATES

For each protein target in a given dataset, functional templates are defined as these proteins that structurally align onto the target with a statistically significant TM (template modeling)-score of ≥0.4 (Zhang and Skolnick, 2004a) and bind their partners in a similar location. Here, we use distance thresholds of 4, 2, 3, 6, and 6 Å for ligands, metal, iron–sulfur clusters, protein, and DNA interfaces, respectively. The distances are measured upon the global template-to-target superposition generated by fr-TM-align, a structure alignment program (Pandit and Skolnick, 2008). The ratio of the number of positives and negatives across ligand-, metal-, Fe/S-, protein-, and DNA-binding proteins is 0.24, 0.10, 0.87, 0.10, and 0.31, respectively. In addition, we also assess the chemical and geometrical conservation of bound molecules, i.e., the template- and target-bound metals are of the same type, the pairwise Tanimoto coefficient (Tanimoto, 1958), *TC*, calculated for Daylight 1024-bit SMILES strings between the template- and target-bound ligands is ≥0.5, or the nucleotide composition of the template- and target-bound DNA is similar (AT- or GC-rich). For protein–protein complexes, we assess the local structural similarity of binding interfaces using iAlign with a significant interfacial score, IS (interface similarity)-score, of ≥0.191 (Gao and Skolnick, 2010). According to these criteria, the positives/negatives ratio for ligand-, metal-, Fe/S-, protein-, and DNA-binding proteins is 0.21, 0.70, 1.17, 0.21, and 1.16, respectively.

### META-THREADING BY *e*THREAD

To identify functional templates, we use *e*Thread (Brylinski and Lingam, 2012), which integrates ten state-of-the-art protein threading/fold recognition algorithms: CSI-BLAST (Biegert and Soding, 2009), COMPASS (Sadreyev and Grishin, 2003), HHpred (Soding, 2005), HMMER (Eddy, 1998), pfTools (Bucher et al., 1996), pGenThreader (Lobley et al., 2009), SAM-T2K (Hughey and Krogh, 1996), SP3 (Zhou and Zhou, 2005), SPARKS2 (Zhou and Zhou, 2005), and Threader (Jones et al., 1992). *e*Thread was originally design to detect structural templates using machine learning and a set of feature vectors composed of individual threading scores. Here, we extend this functionality to include ligand-, metal-, Fe/S-, protein-, and DNA-binding probability estimates. Specifically, for each aspect of molecular function, we constructed two machine learning models with different levels of optimization to assess whether a particular template (1) binds its partners in a similar location, and (2) binds chemically similar molecules and/or the binding mode is similar. We refer to these models as *Location* and *Features*, respectively. We use a Naïve Bayes classifier (NBC) to combine individual threading scoring functions into a single probabilistic score. In this classifier, the real-value attributes are modeled by a Gaussian distribution, i.e., the classifier first estimates a normal distribution for each threading component by computing the mean and standard deviation of the training data in that class, which is then used to estimate the posterior probabilities during classification. Both classifiers, *Location* and *Features*, are independently trained on threading scores. The accuracy of template selection is assessed using twofold cross validation; Pearson’s chi-squared test applied to each individual scoring function confirmed that both subsets are characterized by the same central tendency and dispersion measures as the whole dataset. Thus, considering a maximum sequence identity of 40% between any two dataset proteins, this protocol provides a sufficient cross validation. Furthermore, the imposed pairwise sequence identity threshold automatically excludes close homologs from benchmarks.

### EXAMPLE PROTEINS

We selected the following representative examples: recombinant *A. aegerita* lectin complexed with lactose (PDB-ID: 2zgm), amino acid acyl-carrier protein ligase 1 from *B. japonicum* bound to zinc (PDB-ID: 3mey), NADH-quinone oxidoreductase from *T. thermophiles* complexed with Fe_4_S_4_ (PDB-ID: 2fug), transcription factor PU.1 from mouse bound to DNA (PDB-ID: 1pue), and a homodimer of hypothetical transposase from *S. tokodaii* (PDB-ID: 2ec2). In parentheses are the PDB IDs of weakly homologous (<40% sequence identity) templates identified by *e*Thread for 2zgm (1c1l, 1d2s, 1g86, 1gzw, 1hdk, 1is3, 1kel, 1kjl, 1kjr, 1lhu, 1lhw, 1ngx, 1qfm, 1qkq, 1slt, 1t2q, 1w6o, 1w6p, 2bkl, 2d03, 2d6m, 2eak, 2eal, 2nmo, 2ny1, 2r0h, 2vno, 2wkk, 2wt0, 2wt1, 2xg3, 2z3z, 2zaa, 2zab, 2zac, 2zhk, 2zhl, 2zhm, 3a71, 3a72, 3ap6, 3ap7, 3gal, 3h3l, 3htl, 3nv3, 3nv4, 3o4h, 4gal); 3mey (1b8a, 1e1t, 1e22, 1e24, 1eqr, 1evk, 1evl, 1fyf, 1kog, 1nyq, 1qf6, 1x54, 1x55, 2cim, 2cja, 2i4o, 2j3m, 2q7e, 2q7g, 2rhq, 2rhs, 2xgt, 2xti, 2zcd, 2zce, 2zin, 3a31, 3a74, 3bju, 3e9h, 3e9i, 3nem, 3qtc); 2fug (1cc1, 1e3d, 1fp4, 1frf, 1frv, 1g20, 1g21, 1h1l, 1h2a, 1h2r, 1l5h, 1l9g, 1m1n, 1m1y, 1m34, 1mio, 1n2c, 1qgu, 1qh1, 1qh8, 1ui0, 1ui1, 1vk2, 1yqw, 1yrq, 2a5h, 2afh, 2afi, 2afk, 2d3y, 2ddg, 2dp6, 2frv, 2min, 2wpn, 2xdq, 3aek, 3aet, 3k1a, 3min, 3myr, 3pdi); 1pue (1if1, 1j59, 1k79, 1lb2, 1o3s, 1run, 1t2k, 1xsd, 1yo5, 2cgp, 3e54, 3jtg); and 2ec2 (1a9n, 1hr6, 1y13, 2a6m, 2a6o, 2ar9, 2vic, 2vih, 2vju, 3a4i, 3a74, 3bju, 3lmb).

## RESULTS AND DISCUSSION

We evaluate the performance of *e*Thread in template selection using five datasets that comprise ligand-, metal-, and iron/sulfur-binding proteins as well as protein–protein and protein–DNA complexes. For each target protein, we identify in the PDB library structurally similar proteins that produce statistically significant structure alignment with a TM-score of ≥0.4. These templates that bind molecular partners in similar locations are considered positives with the remaining categorized as negatives. In the subsequent analysis, we examine another requirement for being a positive, which is either a significant chemical similarity of bound molecules for small ligands and metal ions, a similar nucleotide composition for protein–DNA complexes, or a similar interfacial geometry for protein–protein assemblies. The extended version of *e*Thread employs highly tuned machine learning models to provide a set of probability estimates for various aspects of protein molecular function. These are primarily used to assess whether a particular template binds its partners in a similar location; moreover, another set of probabilities estimate whether the template-bound compounds are chemically similar and/or the binding mode between macromolecules is the same. It is important to note that we explore remote evolutionary relationships between protein, excluding those templates that share>40% sequence identity with the target.

**FIGURE 1:**
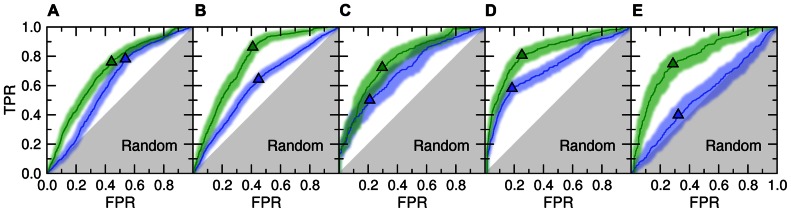
**ROC plots for template identification by meta-threading andmachine learning.** The following aspects of molecular function are considered: **(A)** ligand-, **(B)** metal-, **(C)** Fe/S-, **(D)** protein-, and **(E)** DNA-binding. Two different NBC models are trained to detect functionally related proteins that bind their partners in similar locations (*Location*, green) and bind chemically similar molecules or the binding mode is similar (*Feature*, blue). Triangles depict optimal cut-off points; solid lines and transparent bands are the ROC curve and its non-parametric two-sided confidence bounds, respectively. Gray area corresponds to prediction accuracy no better than random. TPR, true positive rate; FPR, false positive rate.

**Figure 1** shows the accuracy of template identification by *e*Thread for all considered aspects of molecular function. First, we assess the recognition of these templates that bind their partners in similar locations [green ROC (receiver operating characteristic) curves]. The recognition rates are very high, typically 70–80%, at the expense of a relatively low false positive rate with tight 95% confidence bounds (Kestler, 2001). For instance, the true and false positive rate for the selection of weakly homologous Fe/S-binding templates is 0.72 and 0.30, respectively. Many existing approaches to evolution/structure-based binding site prediction cluster the centers of mass of molecules bound to the identified templates upon the global template-to-target superposition (Pons and Labesse, 2009; Roy et al., 2010; Wass et al., 2010). The posterior probabilities estimated by *e*Thread can be used as weight factors in clustering and the subsequent site ranking. This would allow for more precise binding site identification as well as for the improved ranking of predicted binding sites.

Despite the fact that only remote evolutionary relationships between proteins are being explored in the presented benchmarks, meta-threading was found to provide useful insights into the chemical properties of bound molecules and the interfacial geometry; this is shown in **Figure 1** as blue ROC curves. This feature can be used to improve the prediction of the chemical properties of binding ligands and iron–sulfur clusters or the type of binding metal. Here, the most accurate is the recognition of similar protein-binding interfaces with a sensitivity of 58% at only 18% false positive rate. This can be beneficially exploited further to extend the target coverage and to improve the accuracy of approaches to protein docking by interfacial similarity (Sinha et al., 2010). The only aspect of molecular function that cannot be predicted by meta-threading is the nucleotide composition of bound DNA (note that the locations of DNA-binding sites are predicted with a high accuracy). This is due to the fact that many DNA-binding proteins associate with non-specific DNA sequences and slide along to search for the specific DNA target sequence (Halford and Marko, 2004).

The area under ROC is a commonly used method to assess the overall performance of ranking approaches. In **Table 1**, we report the area under the ROC curve for individual threading methods compared to that of *e*Thread. In the majority of cases, the performance of *e*Thread is notably better than that of single-threading algorithms, particularly in the prediction of the chemical properties of binding molecules. The exceptions are HHpred and pfTools in predicting metal-binding sites and binding metals [area under the curve (AUC) of 0.783 and 0.619, respectively], SP3 and SPARKS in detecting DNA-binding aspects (AUC of 0.795 and 0.538, respectively), and COMPASS in recognizing protein–protein interfacial geometry (AUC of 0.732). **Table 2** extends this analysis further by accounting for the so-called “early recognition problem.” By analogy to virtual screening, where one requires active compounds to be ranked as high as possible, template identification approaches also should rank good templates early in an ordered list. BEDROC (Boltzmann-enhanced discrimination of ROC) is a generalization of ROC developed specifically to analyze methods that need to segregate positives toward the front of a rank-ordered list (Truchon and Bayly, 2007). As shown in **Table 2**, except for HHpred in predicting metal-binding sites (BEDROC of 0.723) and DNA feature prediction, which is close to random for all methods, *e*Thread most efficiently tackles the problem of the early recognition of functionally related templates across all binding datasets. These results are finally supported by *Z*-statistics calculated by the Wilcoxon rank-sum (aka Mann–Whitney *U*) test. **Table 3** shows that only SP3 in the identification of DNA-binding sites (*Z*-score of 23.18) gives better *Z*-statistics than *e*Thread.

**Table 1:** Area under the ROC curve for individual threading methods compared to that of *e*Thread.

Method	Ligand-binding		Metal-binding		Fe/S-binding		DNA-binding		Protein-binding	
	*Location*	*Features*	*Location*	*Features*	*Location*	*Features*	*Location*	*Features*	*Location*	*Features*
COMPASS	0.663	0.586	0.695	0.568	0.625	0.597	0.751	0.490	0.814	**0.732**
CSI-BLAST	0.649	0.604	0.732	0.593	0.599	0.566	0.674	0.527	0.757	0.659
HHpred	0.690	0.635	**0.783**	0.617	0.714	0.666	0.774	0.529	0.805	0.698
HMMER	0.656	0.602	0.731	0.595	0.606	0.572	0.734	0.522	0.778	0.718
pfTools	0.652	0.605	0.763	**0.619**	0.583	0.567	0.727	0.519	0.749	0.692
pGenThreader	0.618	0.528	0.671	0.553	0.597	0.564	0.732	0.535	0.771	0.671
SAM-T2K	0.663	0.605	0.758	0.603	0.646	0.595	0.750	0.512	0.786	0.691
SP3	0.674	0.597	0.745	0.596	0.627	0.590	**0.795**	0.532	0.806	0.701
SPARKS2	0.667	0.587	0.738	0.591	0.617	0.576	0.776	**0.538**	0.808	0.704
Threader	0.626	0.540	0.631	0.548	0.610	0.513	0.695	0.511	0.761	0.679
*e*Thread	**0.694**	**0.639**	0.778	0.618	**0.772**	**0.704**	0.781	0.527	**0.819**	0.720

*Location and Features as defined in Section “Methods.” Best performing algorithms are highlighted in bold.*

**Table 2:** BEDROC scores calculated for individual threading methods compared to that of *e*Thread.

Method	Ligand-binding		Metal-binding		Fe/S-binding		DNA-binding		Protein-binding	
	*Location*	*Features*	*Location*	*Features*	*Location*	*Features*	*Location*	*Features*	*Location*	*Features*
COMPASS	0.619	0.535	0.642	0.561	0.587	0.588	0.712	0.505	0.784	**0.712**
CSI-BLAST	0.608	0.551	0.678	0.585	0.565	0.554	0.633	0.525	0.728	0.640
HHpred	0.641	0.575	**0.723**	0.604	0.691	0.664	0.721	0.535	0.774	0.678
HMMER	0.613	0.547	0.676	0.587	0.566	0.556	0.704	0.529	0.745	0.691
pfTools	0.608	0.547	0.702	0.605	0.552	0.555	0.695	0.530	0.713	0.666
pGenThreader	0.580	0.481	0.620	0.547	0.578	0.552	0.698	**0.542**	0.742	0.653
SAM-T2K	0.619	0.551	0.701	0.593	0.613	0.581	0.715	0.520	0.756	0.672
SP3	0.625	0.542	0.685	0.586	0.588	0.577	0.741	0.539	0.774	0.685
SPARKS2	0.620	0.543	0.680	0.581	0.587	0.566	0.742	0.545	0.777	0.688
Threader	0.578	0.486	0.569	0.537	0.599	0.519	0.651	0.520	0.720	0.649
***e*Thread**	**0.646**	**0.579**	0.716	**0.608**	**0.758**	**0.714**	**0.744**	0.537	**0.787**	0.700

*Location and Features as defined in Section “Methods.” Best performing algorithms are highlighted in bold.*

**Table 3:** *Z*-statistics calculated by the Wilcoxon rank-sum test for individual threading methods compared to that of *e*Thread.

Method	Ligand-binding		Metal-binding		Fe/S-binding		DNA-binding		Protein-binding	
	*Location*	*Features*	*Location*	*Features*	*Location*	*Features*	*Location*	*Features*	*Location*	*Features*
COMPASS	11.54	6.94	13.48	6.42	4.36	4.27	17.00	-2.50	20.46	17.52
CSI-BLAST	8.12	1.63	7.59	4.60	2.38	1.36	6.63	-1.42	15.87	15.03
HHpred	13.06	8.55	15.06	7.31	13.43	11.20	18.19	0.16	21.40	17.09
HMMER	8.87	1.37	9.35	5.71	1.17	1.04	17.35	0.88	17.03	14.80
pfTools	12.04	8.75	19.36	9.21	6.05	4.94	17.46	0.65	20.33	16.97
pGenThreader	12.38	6.48	13.92	7.04	8.32	3.07	17.04	**2.91**	21.54	14.45
SAM-T2K	12.76	9.29	18.26	8.42	9.99	6.32	18.70	0.64	22.86	16.75
SP3	14.41	8.87	18.11	8.54	9.69	6.79	**23.18**	1.98	24.23	17.48
SPARKS2	13.79	8.26	17.68	7.62	8.89	5.70	21.61	2.48	24.12	17.96
Threader	10.99	4.86	8.03	5.64	8.46	1.26	15.03	0.59	22.15	14.96
***e*Thread**	**15.37**	**11.63**	**20.39**	**9.85**	**21.17**	**15.62**	21.97	1.41	**25.39**	**19.92**

*Location and Features as defined in Section “Methods.” For each column, the best separation of positives and negatives is highlighted in bold*.

Tables 1,2, and 3 also clearly indicate performance differences between individual methods. For instance, COMPASS, HHpred, and SP3 are the most effective single-threading algorithms in the prediction of protein-, metal-, and DNA-binding sites, respectively. In view of the fact that meta-threading puts much higher demands for high-performance computing resources (Brylinski and Feinstein, 2012), these results provide useful guidelines for selecting a single-threading method with respect to a particular functional aspect when computational speed is critical. Nevertheless, this comparative analysis perspicuously demonstrates that *e*Thread, which uses meta-threading and machine learning models constructed separately for different facets of protein molecular function, systematically outperforms single-threading methods providing higher overall accuracy.

**FIGURE 2:**
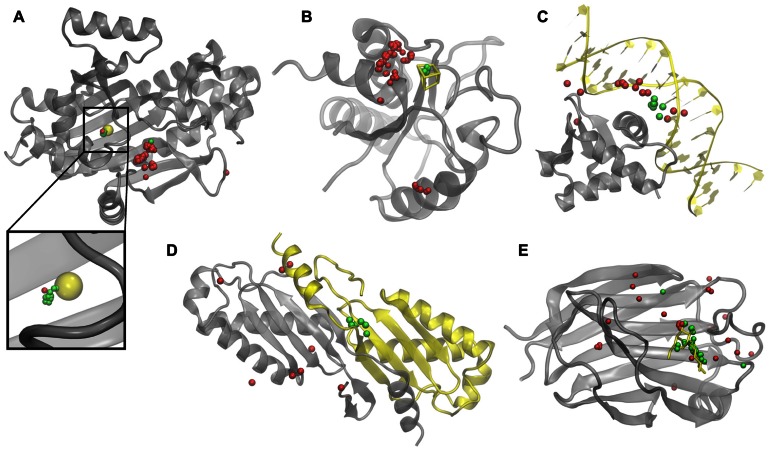
**Examples of binding sites in weakly homologous templates.** Functional templates are identified by *e*Thread for **(A)** aa:CP, **(B)** NDH-1, **(C)**PU.1, **(D)** tnpA, and **(E)** rAAL. Target structures and bound molecular partners are shown in gray and yellow, respectively. Template structures (not shown) are superimposed onto the target; binding sites predicted to be in similar and different locations are colored in green and red, respectively.

To illustrate how weakly homologous templates identified by *e*Thread pick out binding sites, we selected five representative examples, one for each aspect of molecular function: recombinant lectin (rAAL) complexed with lactose, amino acid acyl-carrier protein ligase 1 (aa:CP) bound to zinc, NADH-quinone oxidoreductase (NDH-1) complexed with Fe_4_S_4_, transcription factor PU.1 bound to DNA, and a homodimer of hypothetical transposase (tnpA). In **Figure 2**, upon the global template-to-target superposition, these templates that are predicted to have a binding site in a similar location are shown in green. Red spots indicate template binding sites, which are below the optimal probability threshold, i.e., are predicted to bind molecules in different locations. In all cases, there are very few false positives and false negatives; the consensus amongst templates predicted to bind their molecular partners in a similar location precisely identifies the correct binding site. Note that for aa:CP and NDH-1, a simple majority voting would result in the incorrect prediction of the binding site location.

## CONCLUSION

We extended the functionality of *e*Thread, a recently developed meta-threading approach (Brylinski and Lingam, 2012), to address the problem of identifying weakly homologous templates for function annotation in the “twilight zone” of sequence similarity. This method successfully covers many facets of molecular function including the interactions between proteins and small molecules, metal ions, inorganic clusters, nucleic acids as well as other proteins. It provides not only the location on a protein surface where the interactions take place, but also useful insights into the chemical properties of binding molecules and the geometrical aspects of binding. Such information can be straightforwardly incorporated into any existing evolution/structure-based algorithm for binding site prediction and functional annotation. Moreover, binding pocket prediction can be extended further to include additional downstream analyses, e.g., binding site comparison and mining. For example, Pocket-Surfer (Chikhi et al., 2010) and Patch-Surfer (Sael and Kihara, 2010,^2012^) identify ligand molecules that bind to predicted pockets by comparing their geometrical and physicochemical characteristics with a database of known binding pockets. These algorithms are likely to benefit from the improved accuracy of upstream pocket detection.

*e*Thread is freely available to the community at http://www.brylinski.org/ethread as a webserver and a stand-alone software distribution. The latter can be installed in a local environment for high-throughput computations and the integration with existing structure-based protein function inference pipelines. The documentation includes detailed installation instructions and illustrative examples.

## Supplementary Material

The Supplementary Material for this article can be found online at http://www.frontiersin.org/Bioinformatics_and_Computational_Biology/10.3389/fgene.2013.00118/abstract

## Confilct of interest statement

The author declares that the research was conducted in the absence of any commercial or financial relationships that could be construed as a potential conflict of interest.
